# Cross-reactive, natural IgG recognizing *L. major* promote parasite internalization by dendritic cells and promote protective immunity

**DOI:** 10.1007/s00109-021-02137-4

**Published:** 2021-10-04

**Authors:** Filiz Dermicik, Susanna Lopez Kostka, Stefan Tenzer, Ari Waisman, Esther Von Stebut

**Affiliations:** 1 Institute for Molecular Medicine, University Medical Center, Johannes Gutenberg University Mainz, Mainz, Germany; 2 Department of Dermatology, University Medical Center, Johannes Gutenberg University Mainz, Mainz, Germany; 3 Institute for Immunology, University Medical Center, Johannes Gutenberg University Mainz, Mainz, Germany; 4 Research Center for Immunotherapy (FZI), University Medical Center, Johannes Gutenberg University Mainz, Mainz, Germany; 5 Focus Program in Translational Neuroscience (FTN), University Medical Center, Johannes Gutenberg University Mainz, Mainz, Germany; 6 Department of Dermatology, Faculty of Medicine, University of Cologne, Cologne, Germany

**Keywords:** *Leishmania major*, Dendritic cell, B cell, Natural IgG

## Abstract

**Abstract:**

In cutaneous leishmaniasis, infection of dendritic cells (DC) is essential for generation of T cell-dependent protective immunity. DC acquires *Leishmania major* through Fc receptor (FcR)-mediated uptake of complexes comprising antibodies bound to parasites. We now assessed the development of the initial B cell and DC response to the parasite itself and if natural IgG play a role. *L. major* parasites display large numbers of phospholipids on their surface. *P*arasites were opsonized with normal mouse serum (NMS), or serum containing anti-phospholipid IgG (PL). We found that *L. major* bound to PL which significantly enhanced parasite phagocytosis by DC as compared to NMS. Similar results were obtained with cross-reactive human PL antibodies using myeloid primary human DC. In addition, mice infected with PL-opsonized parasites showed significantly improved disease outcome compared to mice infected with NMS-opsonized parasites. Finally, IgMi mice, which produce membrane-bound IgM only and no secreted antibodies, displayed increased susceptibility to infection as compared to wild types. Interestingly, once NMS was administered to IgMi mice, their phenotype was normalized to that of wild types. Upon incubation with IgG-opsonized parasite (IgG derived from infected mice or using PL antibodies), also the IgMi mice were able to show superior immunity. Our findings suggest that “natural” cross-reactive antibodies (e.g., anti-PL Ab) in NMS bind to pathogens to facilitate phagocytosis, which leads to induction of protective immunity via preferential DC infection. Prior *L. major*-specific B cell-priming does not seem to be absolutely required to facilitate clearance of this important human pathogen in vivo.

**Key messages:**

- We found that anti-phospholipid (anti-PL) antibodies enhance phagocytosis of L. major by DCs.
- We also found that normal mouse sera have natural antibodies that can imitate PL specific antibodies.
- Using different genetically modified mice, we found that these antibodies can be IgG, not only IgM.

## Introduction

Infections with *Leishmania* spp. represent a major burden in endemic countries [1]. Disease manifestation ranges from self-limited cutaneous leishmaniasis to disseminated, recurrent, mucocutaneous, and visceral disease. Untreated, visceral leishmaniasis is a severe threat for the host’s life. A vaccine does not exist yet [1].

Healing and lifelong immunity against this important intracellular pathogen depends on the development of IFN-producing Th1/Tc1 cells, whereas parasite persistence and disease progression is associated with Th2 cell dominance, regulatory T cells, and/or Th17 responses [2, 3]. IFNγ release leads to NO production which eliminates the parasites. Protective immunity is induced by infected dendritic cells (DC). After inoculation of *Leishmania major* into skin by the sandfly, promastigote parasite life forms are ingested by skin-resident macrophages (MΦ) and neutrophils. Within MΦ, parasites transform into non-flagellated amastigote life forms and replicate [3]. Later, released amastigotes are taken up by other host cells, such as DC. Infected DC process parasite antigen, migrate to draining lymph nodes and prime T cells [2]. Release of IL-12 as well as other cytokines from infected DC directs Th1/Tc1 education of parasite-specific T cells [4].

Whereas MΦ utilize complement receptor (CR)3 for parasite uptake [5], in DC, FcγRI/III is responsible for parasite internalization [6]. Interestingly, early on, CR3-associated parasite uptake leads to silencing of the infected MΦ, whereas in established infections, FcγR-mediated uptake of amastigotes by MΦ induces anti-inflammatory IL-10 production promoting Th2/Treg development and parasite persistence. FcγR-mediated parasite uptake in DC, in contrast, induces cell activation, CD4 T cell priming, and also antigen cross-presentation [6]. Therefore, antibody-mediated parasite uptake by DC is important for the development of protection against the parasite. Production of anti-*Leishmania* IgG is thus a prerequisite for efficient (cross-)priming of *Leishmania*-specific Th1/Tc1 cells. In line, in the absence of B cells, disease development was more severe with larger lesion volumes, higher parasite burdens, delayed T cell priming, and reduced IFNΓ production [6]. Previously, we showed that *Leishmania*-specific IgG was present in sera at the time of DC accumulation in lesions [6].

It remains an open question how the initial B cell response to the parasite itself develops in the absence of B cell priming by infected DC. So-called natural antibodies recognizing *Leishmania* spp. [7, 8] may facilitate early parasite internalization by DC. In addition, since parasite membranes contain phosphatidylserine similar to apoptotic bodies, cross-reactive antibodies may play a role. In the present study, we assessed whether anti-phospholipid antibodies generated, e.g., during excessive cell death or natural IgG recognizing *Leishmania* present in nonimmune animals contribute to parasite take DC to promote B cell and T cell priming [9]. We found that anti-phospholipid antibodies from murine or human serum as well as “natural IgG” in normal mouse serum (NMS) bind to *Leishmania* parasites, which is sufficient to promote parasite internalization by DC and promotes better disease outcome in vivo.

## Material and methods

### Animals

Six-to-eight-week-old C57BL/6 mice were purchased from Janvier. B cell-deficient µMT mice were generously provided by Hansjörg Schild, Institute of Immunology, University Medical Center Mainz. IgGi and IgMi mice (both on C57BL/6 background) were described previously [10]. All animals were housed under specific pathogen-free (SPF) conditions in the Translational Animal Research Center (TARC) of the Johannes Gutenberg University, Mainz. All experiments were undertaken with approved license from the Animal Care and Use Committee of the Region Rheinland-Pfalz.

### Parasites and infections

Amastigotes or metacyclic promastigotes of *L. major* clone VI (MHOM/IL/Friedlin) were prepared as previously described [6]. Amastigotes were isolated from infected ears of BALB/c or B cell-deficient µMT mice as described before [11]. Isolated parasites were opsonized with 5% NMS, IMS, or anti-PL for 10 min at 37 °C and washed before in vitro or in vivo infections.

### Sera, IgG enrichment, and parasite binding assay

Normal mouse serum (NMS) was generated from naïve C57BL/6 mice, whereas immune serum (IS) was taken from healed mice that were infected with 2 × 10E^5^ metacyclic promastigotes of *L. major* for ≥ 6 weeks. Antiphospholipid antibody (PL) sera were generated by repeated immunization with apoptotic thymocytes [12]. Before serum was harvested, immunization success was verified by assessing specific IgG binding to apoptotic cells via flow cytometry or in a β-glycoprotein-specific ELISA. Groups of C57BL/6 mice were immunized with recombinant β2-glycoprotein (β2G) in the presence of Freuds adjuvant (FA); serum containing anti- β2G was harvested after 4 weeks. Anti-*Leishmania* IgG ≥ 5–6-week *L. major*-infected BALB/c mice*,* PL- or b2G-specific IgG, was prepared from pooled sera using protein G columns (Pierce Chemical Co.) following the manufacturer’s protocol. Sera were stored at −20 °C before IgG purification. Purified IgG was stored at 4 °C (0.8 mg/mL) in PBS before use.

Normal human serum (NHS) was from healthy control subjects and serum containing anti-*Leishmania* antibodies was obtained from cutaneous leishmaniasis (LM) patients from Marocco. Serum from patients with anti-phospholipid syndrome (PL) was also used.

Parasites (promastigotes or amastigotes) were stained for surface-associated Ig using isotype-specific secondary antibodies reactive with mouse Ig: anti-IgM (Serotec), anti-IgG1 (A85-1), and anti-IgG2a/b (R2-40, all from BD Biosciences). After staining, parasites were washed with PBS/2% BSA, fixed, and analyzed by flow cytometry.

### In vitro stimulation

Bone-marrow-derived DC were generated in RPMI/5% FCS media supplemented with IL-4 (10 µg/mL) and GM-CSF (10 µg/mL) for 6 days as previously described [6]. Cells were harvested on day 6 as immature DC. 2 × 10^5^ DC were co-cultured with opsonized amastigotes from infected BALB/c or µMT mice or cultured with opsonized metacyclic promastigotes (MOI 1:5) for 18h. Afterward, cells were harvested and cytospins were generated as previously described [6]. DiffQuick-stained cells were analyzed for the presence of intracellular and extracellular parasites. At least 200 cells were counted per sample.

### In vivo infections

C57BL/6, µMT, IgGi, and IgMi mice were infected with physiological low dose of 10^3^ metacyclic *L. major* promastigotes in a volume of 10 µL by intradermal injection into both ears using 0.3-mm-diameter needles. In some experiments, parasites were opsonized for 10 min with either NMS, IS, or α-PL and washed. In others, mice were reconstituted i.p. two times with 100 µL of either NMS or IS.

Lesion volumes were measured weekly in three dimensions and are reported as ellipsoids [(a/2 × b/2 × c/2) × 4/3 × π]. Parasites present in lesional tissue were enumerated using a limiting dilution assay as previously described [11].

For measurement of antigen-specific cytokine production, draining LN cells of infected C57BL/6 mice were recovered and single-cell suspensions were prepared. One-million LN cells/200 μL complete RPMI 1640 (Biochrome) were added to 96-well plates in the presence of 25 μg/mL SLA. Supernatants were harvested 48h after stimulation and assayed using ELISAs specific for IFNΓ (R&D Systems), as well as IL-4 and IL-10 (BD).

### Myeloid human DC infected with opsonized parasites

Myeloid human CD1c^+^ DC (hDC) were isolated from peripheral blood according to manufacturer’s instructions using a magnetic cell isolation system and the BDCA-1 human DC isolation kit (Miltenyi, Germany). 1.5 × 10^7^ cells were plated in 2.5 mL RPMI 1640/2% autologous plasma. To enrich purity, cells were washed with warm PBS and finally cultured with X-VIVO 15/plasma (1%) supplemented with recombinant human IL-4 (150 U/mL) and GM-CSF (400 U/mL). Immature hDC were harvested on day 6. 2 × 10^5^ hDC were co-cultured with opsonized amastigotes from infected µMT mice or cultured with opsonized metacyclic promastigotes (MOI 1:5) for 18h. Afterward, cells were harvested and cytospins were generated as previously described. DiffQuick-stained cells were analyzed for the presence of intracellular and extracellular parasites. At least 200 cells were counted per sample.

### Statistical analysis

Statistical analysis was performed using StatView software and unpaired Student’s *t*-test.

## Results

### Antiphospholipid antibody-containing serum binds to *L. major*

Since parasites can enter host cells via a process called “apoptotic mimicry” [13] and because *L. major* life forms express phospholipids on their surface, we generated serum containing antiphospholipid antibodies (PL) using established protocols [12]. To this aim, groups of C57BL/6 mice were infected with *L. major*, on immunized with apoptotic thymocytes in the presence of Freuds adjuvant (FA). As β2-glycoprotein (β2G) is the primary antigen in antiphospholipid syndrome (APS [14]), we also immunized mice with 2G in the presence of FA to obtain serum containing anti-β2G antibodies. As controls, normal mouse serum (NMS) from uninfected C57BL/6 mice and immune serum (IS) from *L. major*-infected mice were used.

**Fig. 1:**
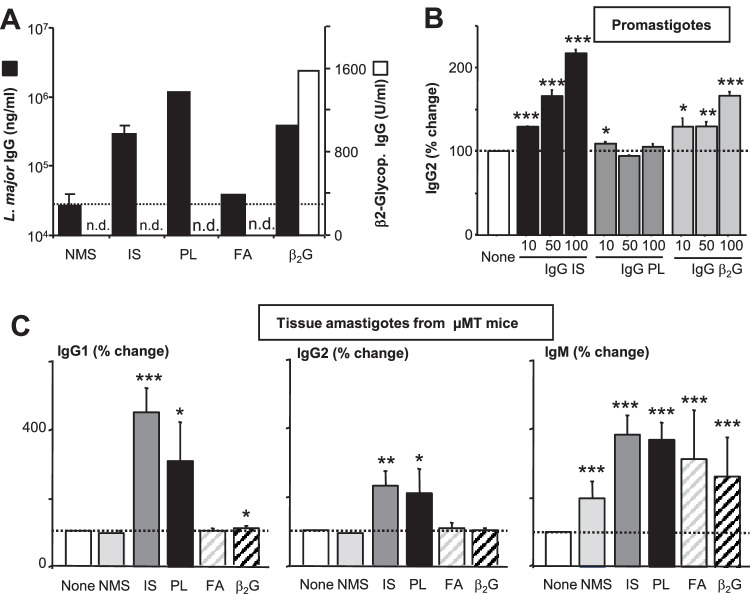
Detection of *L. major* cross-reactive antibodies in serum of mice containing anti-phospholipid antibodies. Serum was obtained from groups of C57BL/6 mice which were left untreated (normal mouse serum, NMS) or which were infected for > 6 weeks with 2 × 10^5^ *L. major* (immune serum, IS). Another group was repeatedly immunized with apoptotic thymocytes (anti-phospholipid Ab, a-PL) or recombinant β2-gycoprotein (a-β2G) in the presence of incomplete Freuds (FA) adjuvant, FA was used as control. **A** Serum was tested for reactivity in an ELISA using *L. major* lysate as substrate or β2-glycoprotein**.** The ELISA were developed using anti-murine IgG. Data are expressed as mean (± SEM, n ≥ 1). **B** Metacyclic promastigotes of *L. major* were obtained from stationary phase cultures (*n* = 2). **C** Amastigotes were prepared from lesions of B cell-deficient µMT mice (*n* = 3). **B** and **C** Parasite preparations were opsonized with different sera (5%, 10 min, 37 °C). Antibody binding was assessed by FACS with anti-mouse secondary antibodies. Binding of Ig was calculated in relation to baseline levels of unopsonized parasites (*p ≤ 0.05, **p ≤ 0.005, and ***p ≤ 0.002)

To test if these different mouse sera from naïve or immunized mice contain antibody that bind to *L. major*, we first assessed antibody binding to parasite antigen by ELISA, using *L. major* promastigote freeze–thaw lysate (soluble *Leishmania* antigen, SLA) as substrate (Fig. 1A, black columns). As standard, we used IgG isolated from the sera of *L. major*-infected mice. Interestingly, IS as well as PL serum contained comparable levels of antibody binding to *Leishmania* lysate, which was 3–4 fold higher compared to binding observed with NMS or sera from mice immunized with adjuvant alone. In addition, serum containing β2G antibodies also bound to SLA. Using an ELISA specific for β2G (white columns), we identified high levels of β2G-IgG in the serum, whereas β2G IgG levels in all other sera were below the detection limit.

Next, we incubated infectious stage metacyclic promastigotes of *L. major* with different concentrations of IgG enriched from IS, PL, or β2G sera. We observed a dose-dependent binding of IS- and β2G-derived IgG with a maximum at 100 ng/mL (Fig. 1B). Binding of PL-derived IgG to promastigotes of *L. major* was not detected.

In addition, we incubated *L. major* amastigotes isolated from lesions of B cell-deficient µMT mice with the different sera for 10 min. Parasites were then extensively washed, and afterward, surface bound antibody was detected by flow cytometry after labeling with PE-conjugated anti-IgG1, anti-IgG2a/b, or anti-IgM (Fig. 1C). As expected, IS contained *Leishmania*-specific IgM, IgG1, and IgG2a/b. We also confirm prior work, as we detected *Leishmania*-specific IgM in the pool of so-called “natural Ab” [15]. Interestingly, we also found IgG and IgM in PL serum that strongly cross-reacted with the surface of *L. major* amastigotes. Anti-β2G-containing IgG antibodies did not bind to *Leishmania*, but the β2G serum contained IgM cross-reacting with amastigote surfaces.

### Cross-reactive antibodies mediate parasite internalization by DC

We next intended to assess if the different IgG antibodies capable of binding to the surface of *L. major* amastigotes are functionally active. Thus, we generated DC using cultures of bone marrow (BM) cells supplemented with GM-CSF and IL-4. BM-DC were harvested as immature cells on day 6 and plated at 2 × 10^5^ cells/mL. Parasite amastigotes derived from infected footpads of wild-type BALB/c or B cell-deficient µMT mice were opsonized with different sera (5 vol%), extensively washed, and then added to DC in a parasite/cell ratio of 5:1. After 18h, cells were harvested, washed, and cytospined.

**Fig. 2:**
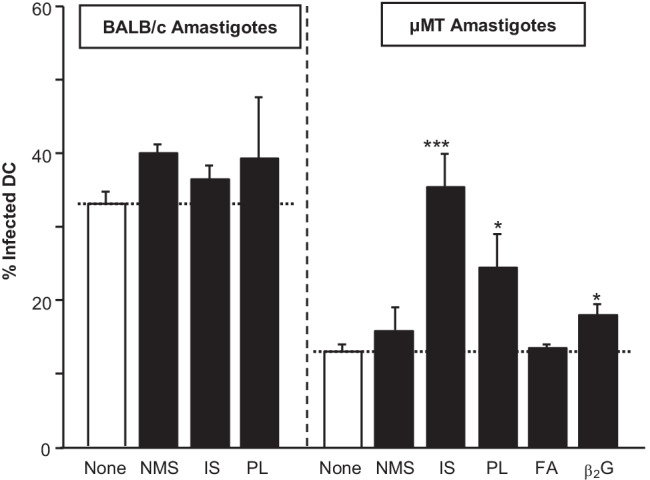
Opsonization of *L. major* with cross-reactive antibodies leads to enhanced parasite uptake by DC. Immature C57BL/6 BMDC (2 × 10^5^) were co-cultured with amastigotes from BALB/c or B cell-deficient µMT mice (1:3). Prior to co-incubation, amastigotes were opsonized with normal mouse serum (NMS), immune serum (IS), serum containing anti-phospholipid antibodies obtained by repeated immunization with apoptotic thymocytes (aPL), or serum containing anti-β2 glycoprotein antibodies (β2G). After 18h, infection rates were determined on cytospins by light microscopy (*n* = 3, *p ≤ 0.05, **p ≤ 0.005, and ***p ≤ 0.002 as compared to unopsonized controls)

Parasite internalization was determined on DiffQuick-stained slides at 100 × (Fig. 2). As described, uptake of parasites isolated from µMT mice was significantly impaired [6]. Pre-incubation with NMS slightly enhanced parasite uptake, whereas incubation with IS led to “normalization” of DC infection rates to levels observed with BALB/c-derived amastigotes. Of note, parasite opsonization with PL also significantly and clearly improved the DC infection rates by ~ 100%. In line with the lower levels of IgG opsonization of amastigotes with b2G-containing serum (compare Fig. 1C), this also enhanced infection rates of DC, but to a lesser extent.

### Cross-reactive anti-phospholipid antibodies improve disease outcome in cutaneous leishmaniasis

**Fig. 3:**
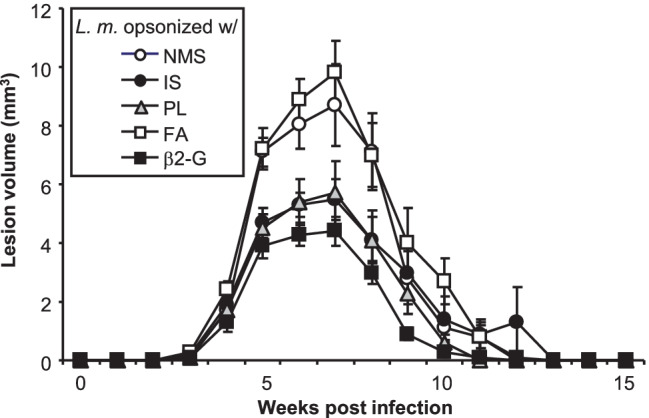
C57BL/6 mice infected with opsonized *L. major* exhibit improved disease outcome. Groups of ≥ 5 C57BL/6 mice were infected with physiological low-dose inocula of *L. major* (10^3^ metacyclic promastigotes). Prior to infection, parasites were opsonized with normal mouse serum (NMS), immune serum (IS), serum containing anti-phospholipid antibodies (a-PL), or — C57BL/6 mice only — serum containing anti-β2 glycoprotein antibodies (β2G) in the presence of Freuds adjuvant (FA). Lesion development was assessed in 3 dimensions weekly and calculated as ellipsoid (mean ± SEM, *p ≤ 0.05, **p ≤ 0.005, and ***p ≤ 0.002 as compared to mice infected with unopsonized parasites, *n* = 10 mice from 2 independent experiments per group)

In subsequent experiments we wanted to assess if parasite opsonization with cross-reactive antibodies alters disease outcome in vivo. To this end, metacyclic promastigotes isolated and enriched from parasite cultures were opsonized with different sera as described above. Parasite opsonized with NMS served as negative control. After extensive parasite washing, groups of five C57BL/6 mice were infected intradermally with 10^3^ *L. major*. Lesion development was monitored for ~ 4 months. In line with prior findings, parasites opsonized with IS induced smaller ear lesions in line with an increased frequency of infected skin DC at early time points [6] (Fig. 3). Surprisingly, parasites opsonized with PL or β2G similarly promoted similarly improved disease outcome as determined by 50% reduced lesion volumes between week 5 and 8 post infection.

**Fig. 4:**
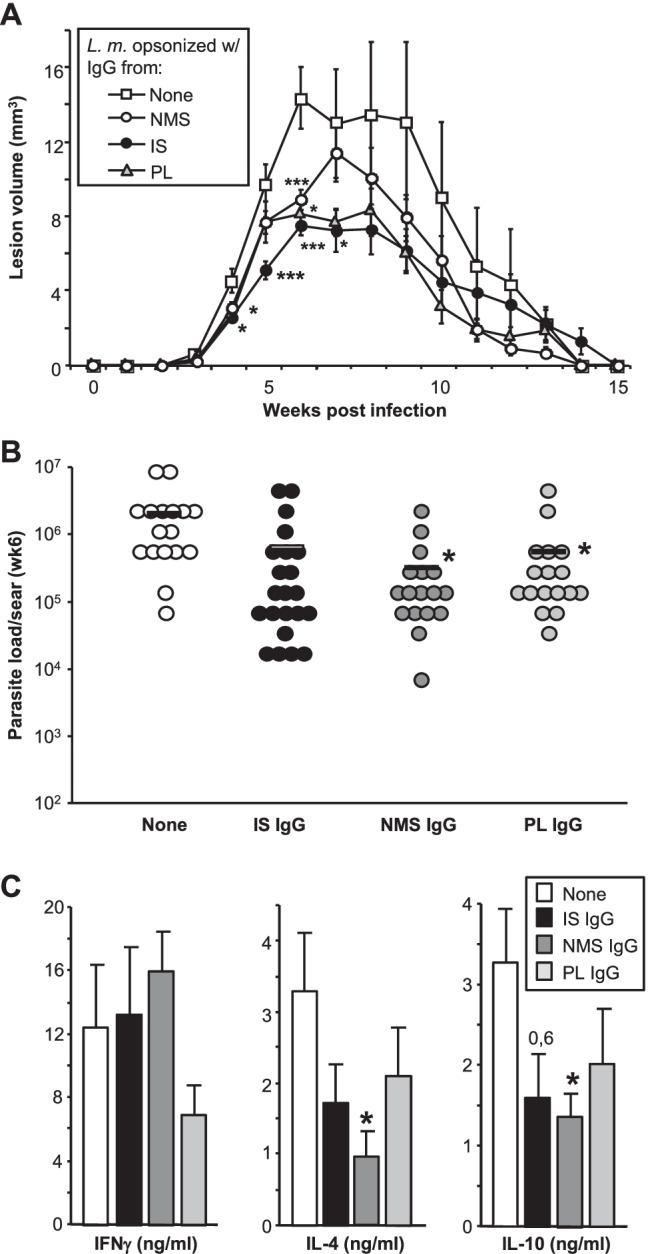
Antibody-opsonized parasites induce efficient immunity in B cell-deficient µMT. Parasites were opsonized with normal mouse serum (NMS), immune serum (IS), serum containing anti-phospholipid antibodies (a-PL), or — C57BL/6 mice only — serum containing anti-β2 glycoprotein antibodies (β2G) in the presence of Freuds adjuvant (FA). Groups of ≥ 5 µMT mice were infected with 10^3^ opsonized metacyclic promastigotes *of L. major*. **A** Lesion development was assessed weekly. **B** In week 6, lesional parasite loads of infected µMT mice were determined by limiting dilution assay. Individual parasite numbers are shown; bars express means. **C** Cytokine profiles of draining µMT LN cells were determined by restimulation with soluble *Leishmania* antigen (SLA). IFNΓ, IL-4, and IL-10 release into 48-h supernatants was assessed by ELISA (mean ± SEM, *n* = 15 mice/group from independent 3 experiments, *p ≤ 0.05, **p ≤ 0.005, and ***p ≤ 0.002 as compared to mice infected with unopsonized parasites)

Next, we used µMT amastigotes incubated with 100 µg IgG enriched from NMS, IS, or PL (Fig. 4). Again, we observed that both IgG from IS or PL resulted in milder disease with smaller lesions, as compared to unopsonized parasites (Fig. 4A). In line, parasite burdens of lesional tissue as determined in week 6 post infection revealed (significantly) smaller parasite burdens in infections initiated in the presence of parasite-binding antibodies (Fig. 4B). Antigen-specific restimulation of draining lymph node cells with SLA revealed unaltered levels of IFN, whereas lower levels of IL-4 and IL-10 were found in those mice infected with IgG-opsonized parasites (Fig. 4C). This is in line with decreased lesion sizes, since prior work showed that parasite elimination in mice is dependent on the activation of infected MΦ by lesional IFN, which is counteracted by IL-4 and — very importantly — IL-10. As such, IL-10 effects on infected MΦ led to parasite persistence [16]. Thus, the ratio between IFN on one hand and Th2/Treg-associated cytokines such as IL-4 and IL-10 appears to be most relevant for disease outcome.

Interestingly, however, in this setting, smaller lesion volumes were also observed, when IgG from NMS was used for opsonization indicating the presence of cross-reactive natural antibodies in serum of naïve mice as well.

### Both Leishmania-specific and cross-reactive antibodies can substitute for genetical lack of IgG

**Fig. 5:**
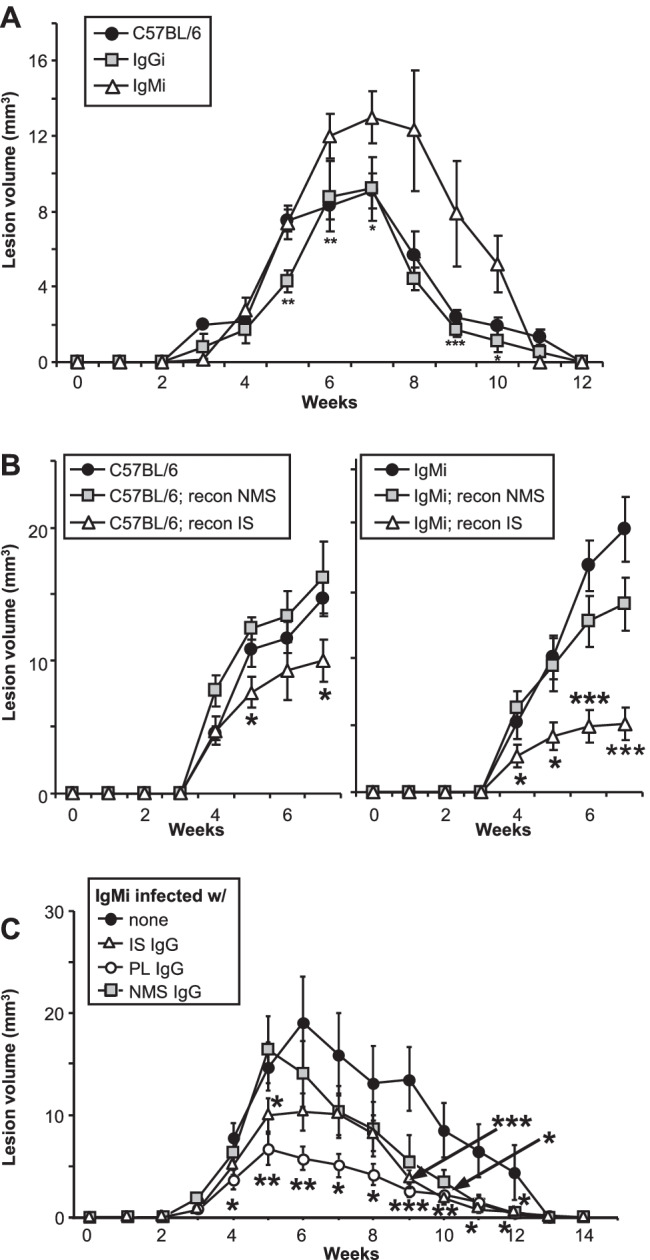
C57BL/6, IgMi, and IgGi mice reconstituted with different sera or infected with different purified IgG opsonized *L. major* exhibit improved disease outcome. **A** Groups of ≥ 5 C57BL/6, C57BL/6 IgMi, or IgGi mice were infected with physiological low-dose inocula of *L. major* (10^3^ metacyclic promastigotes). **B** One week prior to infection, C57BL/6 and B6 IgMi mice were reconstituted i.p. two times with 100 µL of either normal mouse serum (NMS) or immune serum (IS). **C** Prior to infection of IgMi mice, parasites were opsonized with IgG enriched from sera by binding to protein A columns [normal mouse serum (NMS), immune serum (IS), or serum containing anti-phospholipid antibodies (a-PL)]. **A–C** Lesion development was assessed weekly (mean ± SEM, *n* = 10 mice/group from independent 2 experiments, *p ≤ 0.05, **p ≤ 0.005, and ***p ≤ 0.002 as compared to C57BL/6 [**A**], C57BL/6 or IgMi [**B**], or none [**C**])

To better analyze the specific contribution of immunoglobulin subtypes in the response to *Leishmania*, we made use of IgMi and IgG1i mice we have previously generated [17]. IgMi mice are mice where all B cells express IgM, but cannot class switch nor secrete antibodies [17]. In the IgG1i mice, all B cells develop using IgG1 as their B cell receptor, but these cells also cannot class switch, but are able to secrete IgG1 antibodies [17]. We infected IgMi and IgG1i mice on C57BL/6 genetic background with 10^3^ *L. major.* We found that the presence of IgG1 in the sera was sufficient for the mice to mount a full response to the parasite, similarly to the wild-type animals (Fig. 5A). In contrast, absence of secreted antibodies as seen in the IgMi mice was sufficient to delay the recovery of the mice (Fig. 5A).

Next, we reconstituted the IgMi mice or wild-type controls with either NMS or IS and then infected them with *L. major* promastigotes. As expected, in both mouse strains, IS reconstitution induced significantly smaller lesions in vivo confirming that IgG-mediated accelerated parasite uptake by DC promotes improved anti-*Leishmania* immunity (Fig. 5B). Finally, we assessed disease outcome after utilization of opsonized parasites. Prior to infection of IgMi mice, parasites were opsonized with IgG enriched from NMS, IS, or PL serum (Fig. 5C). In line with the experiment shown in Fig. 5B, IS-opsonized parasites facilitated enhanced parasite defense. Again, antiphospholipid antibodies were able to substitute for *Leishmania*-specific IgG from IS in that both IgG antibodies bound to *L. major* promastigotes prior to infection promoted better disease outcome of IgMi mice. IS- and PL-opsonized parasites induced significantly smaller lesion volumes and earlier healing as compared to unopsonized parasites.

Importantly, administration of NMS to IgMi mice “normalized” their phenotype to disease outcome observed in C57BL/6 mice (Fig. 5B). These findings are in line with our data shown above suggesting that NMS also contains a cross-reactive antibody IgG isotype that is capable of binding to parasites with subsequent functional consequences in vitro and in vivo. Further confirmation comes from IgMi mice infected with NMS-coated parasites, which were also able to better control infection (Fig. 5C).

### Human serum contains functionally active, parasite cross-reactive antibodies

**Fig. 6:**
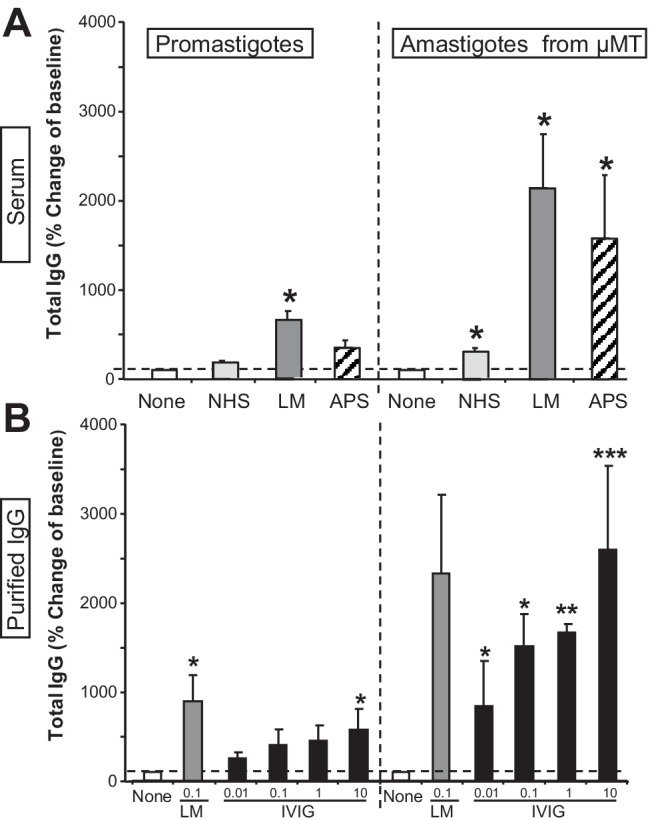
Antiphospholipid antibodies in human sera cross react with *L. major* surface molecules. Metacyclic promastigotes from stationary phase cultures or lesional amastigotes of *L. major* isolated from µMT mice were opsonized with different types of human sera (5%) or purified IgG. **A** Normal human sera (NHS) were obtained from healthy control subjects. Sera from patients with proven cutaneous leishmaniasis (LM, acquired in Marocco, *n* = 4) and from patients with anti-phospholipid syndrome (PL, *n* = 3) were also used. **B** IgG was enriched from LM sera using protein A columns (*n* = 3). Different concentration of intravenous immunoglobulins (IVIG, *n* = 2) were used (µg/mL). **A** and **B** Parasites were analyzed for surface binding of total human Ig using flow cytometry. Binding of Ig was calculated in relation to baseline levels of unopsonized parasites (mean ± SEM, *p ≤ 0.05, **p ≤ 0.005, and ***p ≤ 0.002)

To test the relevance of our findings for humans, promastigotes or amastigotes isolated from B cell-deficient µMT mice were opsonized with normal human serum (NHS), serum from *L. major*-infected patients (LM), or serum from APS patients (APS). Surface-bound IgG on parasites was determined using flow cytometry. Interestingly, LM serum bound weakly to surfaces of promastigotes, but strongly to amastigotes. Interestingly, APS serum contained similar levels of IgG cross-reactive with parasite surfaces (Fig. 6A).

IgG was next enriched from LM sera using protein A columns (Fig. 6B). This and different concentration of intravenous immunoglobulins (IVIG) used for treatment of various (immune) disorders of patients were next used for parasite opsonization prior to flow cytometry. *Leishmania* IgG significantly bound to promastigotes and amastigotes of *L. major*. Interestingly, commercially available IVIG contained IgG that cross-reacted both with promastigote as well as amastigote parasite preparations.

**Fig. 7:**
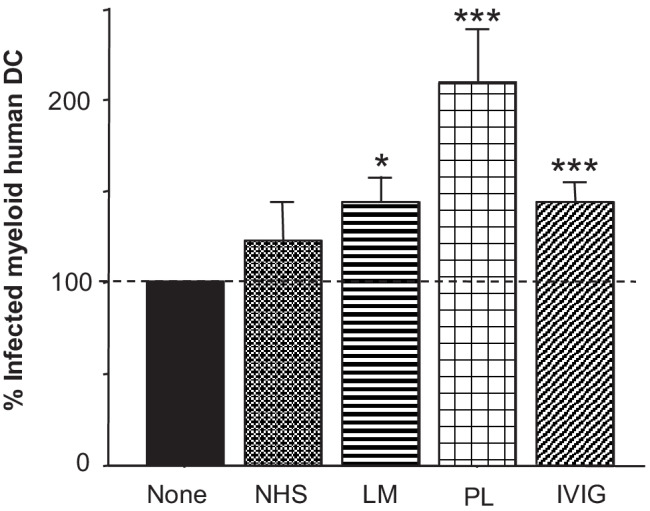
Primary myeloid DC from human blood preferentially internalize parasites with cross-reactive antiphospholipid IgG bound to their surface. CD1a^+^ myeloid human DC were isolated from buffy coats using MACS beads and plated at 2 × 10^5^ cells/mL. Amastigotes isolated from µMT mice were incubated with 5% serum or 10 µg/mL IVIG prior to coincubation with DC (1:3). After 18h, infection rates were determined on cytospins by light microscopy (*n* = 3, *p ≤ 0.05, and ***p ≤ 0.002 as compared to unopsonized controls)

Finally, CD1c^+^ primary myeloid human DC from buffy coats were incubated with µMT amastigotes of *L. major* incubated with LM serum or serum from APS patients or IVIG (MOI 3; Fig. 7). Confirming our murine data using human cells we observed that the presence of IgG (cross-reactive or *Leishmania*-specific) on *Leishmania* surfaces is sufficient to promote enhanced parasite internalization by DC. APS serum increased the percentage of infected DC by almost 100%, whereas LM serum and IVIG enhanced parasite uptake by ~ 30% compared to unopsonized parasites. Interestingly, NMS serum also contained parasite-reactive IgG promoting parasite internalization.

## Discussion

Healing from cutaneous leishmaniasis requires efficient T cell priming and IFN release, both of which depend on infected DC to present antigen to naïve T cells. Unlike CR3-dependent phagocytosis by MΦ, we have shown that parasite internalization by DC occurs via FcγRI and FcγRIII, which can substitute for each other [6]. As such, Fcγ-, FcγRI/FcγRIII-deficient mice, and B cell-deficient µMT mice (all on C57BL/6 background) developed larger lesions and delayed healing, when compared to wild-type mice, due to impaired DC-dependent T cell priming. Our present study confirms this prior observation, but in addition, our data using IgMi and IgGi mice also suggest that indeed secreted antibodies, and not the mere presence of B cells, are critical to mount an effective response to the parasite. As such, similar to B cell-deficient mice, IgMi mice with B cells incapable of producing IgG showed larger lesions sizes compared to the IgGi mice or wild-type mice.

Because both NMS and IS-opsonized amastigotes were taken up to similar extents and NMS-opsonized promastigotes were not phagocytosed by DCs (with efficient binding of IgM to their surface), we previously concluded that IgM is not required for parasite uptake [6]. In the present study we were able to confirm these findings by using normal mouse sera in mice that either lack B cells all together or only secreted antibodies. Further, we were able to show that IgG1-containing sera were able to enhance the entry of amastigotes to both human and mouse DC.

It remained unresolved how the initial B cell response develops in the absence of infected DC. One possibility was that in the pool of “natural” IgG, cross-reactive antibodies may be able to recognize parasites and serve as substitute for the *Leishmania*-specific antibodies that develop later on (rising between week 4 and week 6 post infection [6]). In addition to antigen-specific antibodies, so-called natural antibodies are produced by B-1 cells in both mice and humans after birth [18]. Some have the ability to recognize self-antigens; for a long time it was believed they lack specificity for foreign antigens. Later, data provided evidence for natural IgM recognize diverse microbial antigens and contribute to pathogen elimination [18]. Recently, however, several studies revealed that natural IgG plays a role in innate immunity as well. It was shown to interact with pathogen-associated lectins (e.g., MBL), and to form immune complexes to clear the pathogens [19]. Pathogen-bound natural IgG is also expected to interact with other PRRs, such as C1q, to activate the classical complement pathway [20]. It was shown that natural IgG specifically collaborates with pathogen-associated lectins to elicit an effective antimicrobial action against opportunistic *Pseudomonas aeruginosa* and *Staphylococcus aureus* infection [21].

During apoptotic cell death, phosphatidylserine (PS) exposure on apoptotic bodies is observed. Host recognition of PS on the surface of dying or dead cells via antibodies is an important step in their clearance [9]. In a process called “apoptotic mimicry,” *Leishmania* parasites are known to also expose PS on their own surface serving as “eat-me” signal for phagocytic cells, similar to what is seen by apoptotic cells. Furthermore, some of the infectious-stage promastigotes die (without involvement of caspases) and expose PS themselves. Obligate intracellular amastigote life forms, however, all expose PS on their surface allowing them to enter host cells efficiently [9, 22]. We now assessed whether artificially induced PL antibodies are capable of recognizing *Leishmania* parasites and whether these are functionally active to facilitate parasite entry into relevant host cells. We observed that murine and human anti-phospholipid antibodies bind *Leishmania* life forms. Using different approaches, we identified cross-reactive antibody in PL serum that appear to preferentially bind to surface phospholipids of *L. major*. Interestingly, even though promastigote parasites also express these phospholipids as demonstrated using soluble parasite antigen from this parasite life form in an ELISA, surface phospholipids appear to be more strongly exposed on amastigotes [9, 22]. In addition, we showed that this IgG opsonization was sufficient to promote parasite uptake by DC. Importantly, in vivo, PL-opsonized parasites induced an improved disease outcome with smaller lesion volumes and earlier lesion resolution. Thus, in line with prior observations from our group [6], due to enhanced DC infection in the presence of (cross-reactive) IgG on the parasites, a preferential induction of Th1/Tc1 cells was induced associated with decreased numbers of Th2 and Treg cells, and thus better disease outcome.

Interestingly, we found that parasites opsonized with NMS promoted better disease outcome compared to unopsonized parasites indicating the presence of natural IgG in NMS that is able to recognize *Leishmania*. This was best visible in IgMi (devoid of B cells capable to produce any IgG) substituted with NMS. Here, the clearly larger lesion sizes of IgMi mice were reduced to that of wild-type C57BL/6 mice (Fig. 5). However, the presence of natural, *Leishmania* cross-reactive IgG was also observed in wild-type mice infected with NMS-opsonized parasites compared to unopsonized parasites. The specificity of these natural IgG in murine and human serum is unclear to date. It is tempting to speculate that these are also antibodies recognizing PS on parasite surfaces since they preferentially bind to amastigotes with known higher exposure of PS compared to promastigotes as well.

We found that serum of healthy human control subjects (from a region where leishmaniasis is only reported as travel disease and no history of former infection) also contained IgG capable of binding to *L. major* amastigotes and promoting enhanced parasite uptake by DC. It is important to note that the IVIG used for our study is prepared within Germany (non-endemic for leishmaniasis). Interestingly, enriched human IgG from IVIG from *Leishmania*-negative individuals thus contained IgG that was capable of binding both promastigotes and (probably due to higher PS exposure) *L. major* amastigotes. In line, serum of APS patients with high levels of antiphospholipid antibodies contained IgG that preferentially bound to amastigotes, lesser to promastigotes. Both IgG from APS sera as well as IgG from IVIG promoted enhanced parasite uptake by primary human DC to a degree comparable (or even better in the case of PL-containing APS sera) to immune serum-derived IgG from leishmaniasis patients.

In summary, our data show that normal mouse as well as human sera, even taken from individuals never exposed to *Leishmania,* contain IgG antibodies that are capable of binding to this parasite, facilitate its engulfment by DCs, and enhance a protective immune response of the host. Even though the *Leishmania* antigen(s) recognized is not fully clear, some of the reactivity may be directed against phospholipids more abundant on amastigote, compared to promastigote surfaces. Our findings may pave a way to design better therapy to this parasite and can even serve as first-line precaution for individuals that travel to *Leishmania*-infected areas.

## Data Availability

Not applicable.
